# Antibody Derived Peptides for Detection of Ebola Virus Glycoprotein

**DOI:** 10.1371/journal.pone.0135859

**Published:** 2015-10-21

**Authors:** Luis Mario Rodríguez-Martínez, Alan Roberto Marquez-Ipiña, Felipe López-Pacheco, Roberto Pérez-Chavarría, Juan Carlos González-Vázquez, Everardo González-González, Grissel Trujillo-de Santiago, César Alejandro Ponce-Ponce de León, Yu Shrike Zhang, Mehmet Remzi Dokmeci, Ali Khademhosseini, Mario Moisés Alvarez

**Affiliations:** 1 Centro de Biotecnología-FEMSA, Tecnológico de Monterrey at Monterrey, Monterrey, Nuevo León, México; 2 Biomaterials Innovation Research Center, Brigham and Women's Hospital, Harvard Medical School, Boston, Massachusetts, United States of America; 3 Harvard-Massachusetts Institute of Technology Division of Health Sciences and Technology, Massachusetts Institute of Technology, Cambridge, Massachusetts, United States of America; 4 Wyss Institute for Biologically Inspired Engineering, Harvard University, Boston, Massachusetts, United States of America; 5 Department of Physics, King Abdulaziz University, Jeddah, Saudi Arabia; The Ohio State University, UNITED STATES

## Abstract

**Background:**

Current Ebola virus (EBOV) detection methods are costly and impractical for epidemic scenarios. Different immune-based assays have been reported for the detection and quantification of Ebola virus (EBOV) proteins. In particular, several monoclonal antibodies (mAbs) have been described that bind the capsid glycoprotein (GP) of EBOV GP. However, the currently available platforms for the design and production of full-length mAbs are cumbersome and costly. The use of antibody fragments, rather than full-length antibodies, might represent a cost-effective alternative for the development of diagnostic and possibly even therapeutic alternatives for EBOV.

**Methods/Principal Findings:**

We report the design and expression of three recombinant anti-GP mAb fragments in *Escherichia coli* cultures. These fragments contained the heavy and light variable portions of the three well-studied anti-GP full-length mAbs 13C6, 13F6, and KZ52, and are consequently named scFv-13C6, scFv-13F6, and Fab-KZ52, respectively. All three fragments exhibited specific anti-GP binding activity in ELISA experiments comparable to that of full-length anti-GP antibodies (i.e., the same order of magnitude) and they are easily and economically produced in bacterial cultures.

**Conclusion/Significance:**

Antibody fragments might represent a useful, effective, and low cost alternative to full-length antibodies in Ebola related capture and diagnostics applications.

## Introduction

The recent Ebola outbreak that began in West Africa in December 2013 [1] has revealed how poorly prepared the medical world is to effectively face this disease [2]. As of March 19th 2015, more than 24,600 cases have been documented in West Africa [3]. This implies a great economic and logistic burden.

Current methods to diagnose the presence of the EBOLA virus (EBOV) in biological samples rely mainly on PCR [4–7]. These methods are able to detect EBOV at low viral loads with high accuracy and reproducibility, but they require special instrumentation and trained personnel, which impose heavy restrictions for use of PCR in Ebola epidemic scenarios. Other Ebola diagnostic alternatives include the use of immunological methods based on polyclonal or monoclonal antibodies (mAbs) [8–11]. However, the production of full-length antibodies is a complex process from an engineering perspective, and the currently available production platforms are not sufficiently effective to provide the required rapid response in an emergency.

Antibody fragments present several potential advantages over the use of full-length mAbs [12]. They can be expressed easily and produced readily and economically in bacterial cultures (i.e., *Escherichia coli* cultures) in large quantities. To produce 1 g of a mAb fragment in a bacterial system would cost between 1/10 to 1/100 of the cost of producing 1 g of a full length mAb in a CHO cell system [13–15], the preferred production platform for mAbs. Furthermore, novel expression and purification technologies [16] have greatly simplified the purification of recombinant proteins produced by *E*. *coli*; this purification was traditionally considered a serious drawback of this widely-used expression system [17].

Antibody fragments contain the variable regions of a full-length antibody and conceptually they retain the binding specificity of full-length antibodies. A general decrease in binding affinity of antibody fragments, compared to binding of intact antibodies, has been documented [18,19]; however, the fragment molecules can be tailored by genetic engineering to improve their affinity and stability [20–22]. Many other design adjustments are possible for antibody fragments with respect to size, pharmacokinetics, immunogenicity, specificity, and even effector functions [12,23,24]. The use of antibody fragments to diagnose the presence of viral particles has been recommended and reported in the context of a number of viral diseases [25–27]. A properly optimized mAb fragment should be as effective as a full length mAb in terms of molecular affinity for an ex-vivo diagnostic application. Since a fragment molecule is much smaller than its corresponding mAb (approximately one sixth of the mAb mass), it can be argued that the use of an optimized mAb implies 6X higher binding efficacy per unit of mass.

In this paper, we aimed to produce three antibody fragments that contain variable regions of mAb 13C6, 13F6, and KZ52—three well-studied anti-EBOV full-length mAbs [28]. Briefly, mAb 13C6 is one of the three full-length mAbs contained in M–003 (from Mapp Biopharmaceuticals), a formulation with proven protection against a lethal EBOV challenge in non-human primates [29,30,31]. Full-length mAb 13C6 is also a constituent in Zmapp, the mAb cocktail recently administered to human patients with apparent good results [32]. Mab 13C6 binds the glycan cap region of the glycoprotein (GP) [33]. MAb 13F6 is one of the constituents of ZMab, another anti-EBOV full-length mAb cocktail from Mapp Biopharmaceuticals [34,35]. It binds the mucin-like domain (MLD) in GP, a highly glycosylated region that is cleaved upon the internalization of viral particles into the host cells [33]. KZ52 is a full-length mAb originally isolated from an Ebola disease (EBD) survivor. The 3D structure of this mAb bound to trimeric GP was characterized in detail by Lee et al. [36] and Lee and Saphire [37].

The three antibody fragments that we designed and produced are Fab-KZ52, scFv-13C6, and scFv-13F6, respectively named after their full-length counterparts, mAb KZ52, mAb 13C6, and mAb 13F6. All three fragments were expressed in *Escherichia coli* and produced using straightforward fed-batch culture protocols in instrumented 2.0L reactors. We demonstrate the use of these antibody fragments to capture GP in ELISA experiments.

## Materials and Methods

### Molecular engineering of antibody fragments

**Fig 1:**
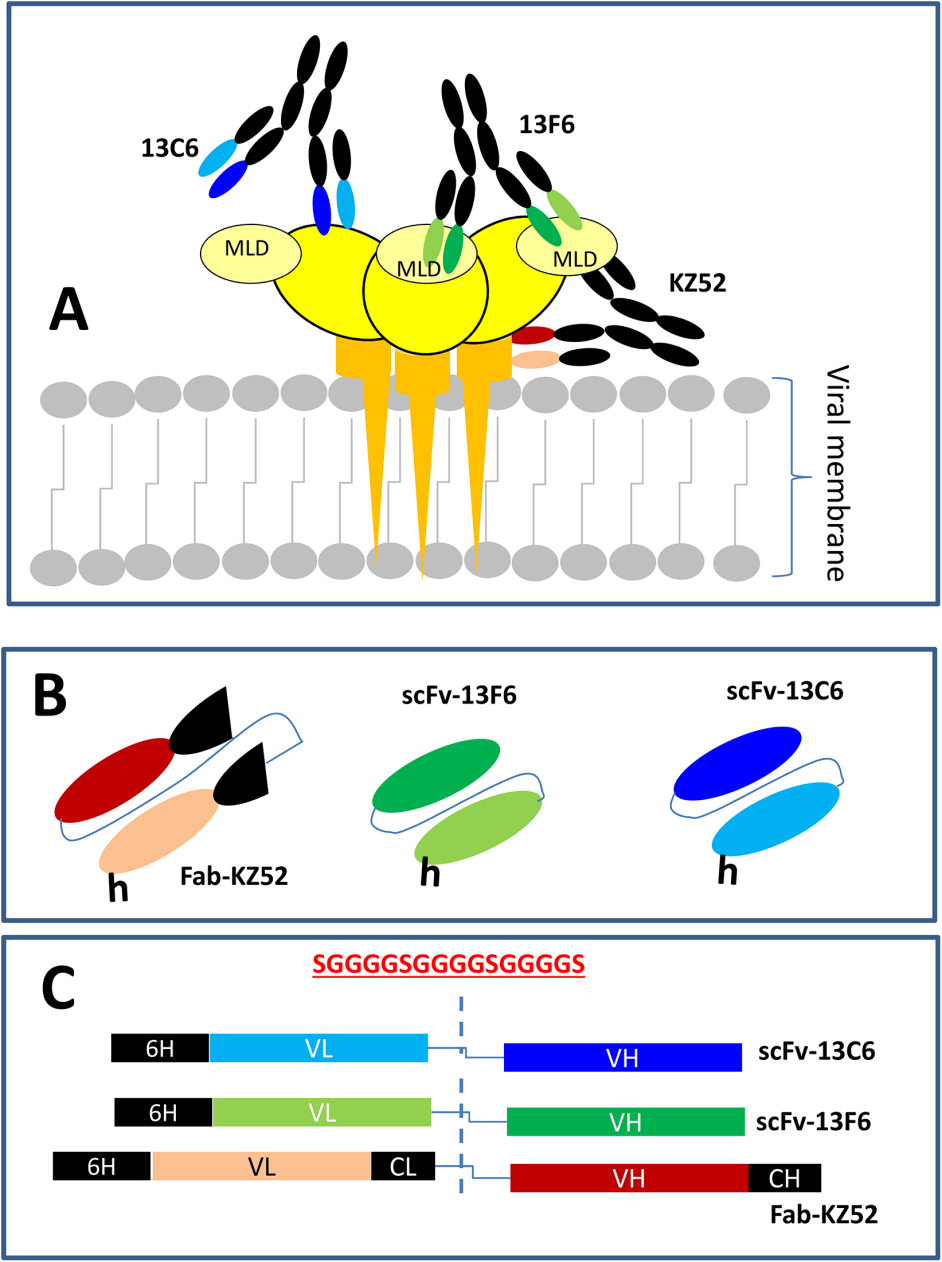
Anti-GP full-length mAbs and their corresponding mAb fragments. (A) The full-length mAbs, 13C6, 13F6, and KZ52, have been widely studied in previous literature.^28-34^ Each of these mAbs targets epitopes in different regions of the GP protein of the Ebola virus (EBOV). In its native form, GP is a trimer composed of three monomers. Each monomer is composed of a GP1 subunit (yellow ovals) and a GP2 subunit (orange stalk). MAb13F6 binds a linear epitope region located in the mucin-like domain (MLD) region of GP^31^ (light yellow small oval). The specific binding region for mAb13C6 is known to be located within the GP1 region^31^ (yellow oval). The binding region of mAbKZ52 has been well characterized;^33,34^ it comprises residues at GP1 (yellow oval) and GP2 (orange stalk). (B) Antibody fragments scFv-13C6, scFv-13F6, and Fab-KZ52 contained the variable regions (responsible for specific GP recognition) of the full-length mAbs that their design was based upon. (C) Schematic representation of the DNA construct used for the expression of scFv-13C6, scFv-13F6, and Fab-KZ52. The sequence of the linker peptide is shown in red.

Three anti GP(EBOV) antibody fragments, inspired by the full-length mAbs 13C6, 13F6, and KZ52, were designed *in silico* and expressed in *E*. *coli* cultures Fig 1A and 1B). Briefly, we designed a DNA construct for each mAb fragment that contained the light variable region (LV) and heavy variable region (HV) of its corresponding mAb (13C6, 13F6, and KZ52). The LV and HV regions were connected by a glycine-serine linker. Each construct (Fig 1C) included a region encoding an N-terminus 6xHisTag to facilitate purification using a Ni^+2^-IMAC column (Fig 1C). In the case of Fab-KZ52, a small portion of the light constant region and the heavy constant region of mAb KZ52 were included in the corresponding construct (Table 1). Constructs were optimized for *E*. *coli* expression and synthetized by our colleagues at DNA 2.0 (San Francisco, CA); they were built into the plasmid pD444-SR and cloned into the BL21 C41(DE3)pLysS strain (for Fab-KZ52) and BL21 C43(DE3)pLysS strain (for scFv-13C6 and scFv-13F6).

**Table 1:** Sequence of the antibody fragments synthesized and studied in this report. Sequences are based in structure information available in literature for each corresponding full length mAb [31,35,36].

13C6
mHHHHHHDDDDKDIVMTQSQKFMSTSVGDRVSLTCKASQNVGTAVAWYQQKPGQSPKLLIYSASNRYTGVPDRFTGSGSGTDFTLTISNMQSEDLADYFCQQYSSYPLTFGAGTKLELRRADAAPTVSIFPPS**SGGGGSGGGGSGGGGS**QLTLKESGPGILKPSQTLSLTCSLSGFSLSTSGVGVGWFRQPSGKGLEWLALIWWDDDKYYNPSLKSQLSISKDFSRNQVFLKISNVDIADTATYYCARRDPFGYDNAMGYWGQGTSVTVS SAKTTAPPVYPLVPGSL
13F6
mHHHHHHDDDDKFSQLVLTQSSSASFSLGASAKLTCTLSRQHSTYTIEWYQQQPLKPPRYVMELKKDGSHSTGDGIPDRFSGSSSGADRYLSISNIQPEDEAIYICGVGDTIKEQFVYVFGGGTKVTVLGQPKSTPTLTVFPPSSEELKENKATLVCLISNFSPSGVTVAWKANGTPITQGVDTSNPTKEGNKFMA**SGGGGSGGGGSGGGGS**CEVQVVESGGGLVKPGGSLKLSCAASGFAFSSYDMSWVRQTPEKRLEWVAYISRGGGYTYYPDTVKGRFTISRDNAKNTLYLQMSSLKSEDTAMYYCSRHIYYGSSHYYAMDYWGQGTSVTV SSAKTTAPPVYPLAPGSL
KZ52
mHHHHHHDDDDKELVMTQSPDSLAVSLGERATINCKSSQSVLYSSNNKSYLAWYQQKPGQPPKLLIYWASTRESGVPDRFSGSGSGTDFTLTISSLQAEDVAVYYCQQYYSAPLTFGGGTKVEIKRTVAAPSVFIFPPSDEQLKSGTASVVCLLNNFYPREAKVQWKVDNALQSGNSQESVTEQDSKDSTYSLSSTLTLSKADYEKHKVYACEVTHQGLRSPVTKSFNR**SGGGGSGGGGSGGGGS**EVQLLESGGGLVKPGGSLRLSCAASGFTLINYRMNWVRQAPGKGLEWVSSISSSSSYIHYADSVKGRFTISRDNAENSLYLQMNSLRAEDTAVYYCVREGPRATGYSMADVFDIWGQGTMVTVSSASTKGPSVFPLAPSSKSTSGGTAALGCLVKDYFPEPVTVSWNSGALTSGVHTFPAVLQSSGLYSLSSVVTVPSSSLGTQTYICNVNHKPSNTKVDKKVEPK

### Production and purification of mAb fragments

**Fig 2:**
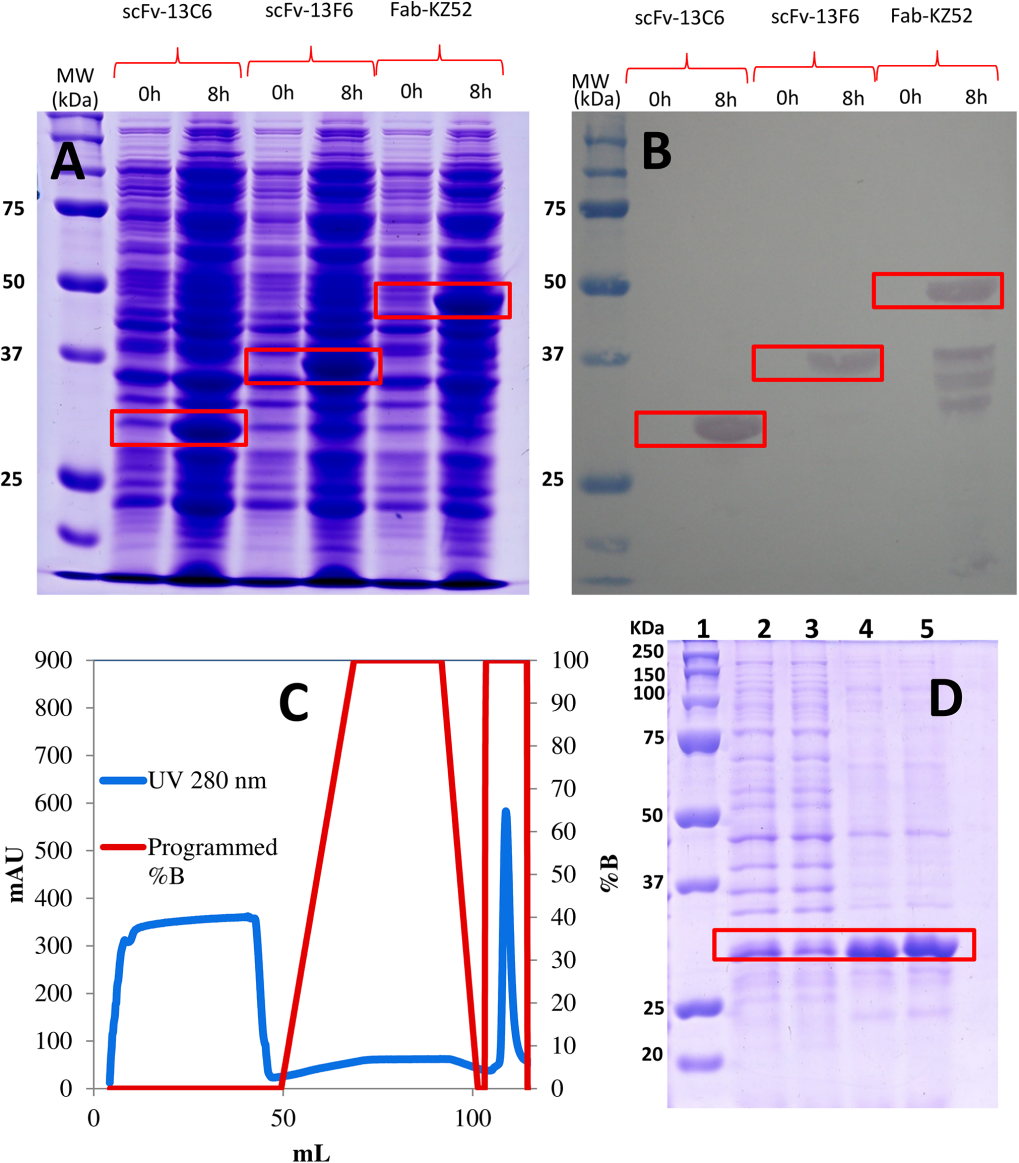
Production of antibody fragments in *E*. *coli* cultures. (A) SDS-Page gel showing the protein expression profile in *E*. *coli* cultures at 0 and 8 hours after IPTG induction. The bands corresponding to each antibody fragment (scFv-13C6, scFv-13F6, and Fab-KZ52) are indicated in red. (B) Corresponding western-blot gel indicating the bands of each anti-body fragments marked with anti-histidine antibodies. (C) Antibody fragments (in this case scFv-13C6) were recovered by immobilized metal-affinity chromatography (IMAC) with 1 mL His Trap FF (GE Healthcare, UK) column pre-charged with Ni^2+^. The absorbance signal at 280 nm, associated with the presence of different protein fractions, is indicated with a blue line; the peak at 100–110 mL of volume (along the blue line) corresponds to fragment scFv-13C6.The percentage of elution buffer fed at different times during the separation protocol is indicated by the red line. (D) SDS-Page gel showing the protein profile in streams at different stages of the IMAC purification process: inlet stream of solubilized inclusion bodies (lane 2), column flow-through stream (lane 3), eluate (lane 4), and purified product solubilized in a 50% glycerol solution (lane 5). The molecular weight ladder is shown in lane 1.

*E*. *coli* was cultured in Luria-Bertani medium with ampicillin (LB-Amp) (supplemented with 15 g/L glucose, 5 g/L potassium phosphate dibasic, 2.5 g/L magnesium sulfate, and 1 mL/L trace nutrients: zinc chloride (2 g/L), cobalt chloride (2 g/L), sodium molybdate (2 g/L), calcium chloride (1 g/L), boric acid (0.5 g/L), and hydrochloric acid (100 mL/L)] in fully instrumented 2-L bioreactors (APPLIKON, Netherlands). The culture conditions were pH = 7.2, 37°C and 400 RPM during growth phase, and 30°C, 600 RPM after induction. Protein production was induced using 1mM isopropyl thiogalactoside (IPTG) approximately 4 hours after initiation of the process. The presence of protein was confirmed with SDS-PAGE and anti-HisTag western blotting (Fig 2).

Cells were recovered by centrifugation in a Z 36 HK (Hermle Labortechnik, Germany) fixed angle rotor centrifuge at 5,000 x g, 4°C, for 15 min. and resuspended in PBS buffer (20 mM PBS, 100 mM NaCl, pH 7.4) at a ratio of 7.5 mL of buffer per 1 g of biomass (wet weight). Cells were disrupted with an Emulsiflex C–3 (Avestin, Canada) high-pressure homogenizer. A protocol of three cycles was followed. The first cycle was set at 5,000 psi; the following two cycles were performed at 20,000 psi.

Cell lysates were centrifuged in a Z 36 HK (Hermle Labortechnik, Germany) centrifuge at 15,000 x g, 4°C, for 30 min. The pellet was resuspended in inclusion bodies IB wash buffer (20 mM PBS, 500 mM NaCl, 1 mM ethylenediaminetetraacetic acid, 2 M urea, 2% v/v Triton X–100, pH 7.4) at a ratio of 25 mL of buffer per 1 g of pellet (wet weight). The suspension was washed vigorously for 30 minutes at room temperature. A final wash with PBS buffer was implemented. The resulting pellet was re-suspended in solubilization buffer (20 mM PBS, 500 mM NaCl, 6 M urea, 1 mM dithiothreitol, 10 mM imidazole, pH 8.2), stirred vigorously at room temperature overnight, and then centrifuged at 15,000 x g, 4°C for 30 minutes. The supernatant containing solubilized IBs was microfiltered through a 0.45 μm membrane (Pall Corporation, NY) and stored at 4°C.

Purified mAb fragment solutions were obtained by immobilized metal-affinity chromatography (IMAC) with 1 mL His Trap FF (GE Healthcare, UK) column pre-charged with Ni^2+^using an Äkta Explorer 100 (GE Healthcare, UK) chromatography system. On-column protein refolding was achieved through the elimination of the chaotropic agent using a 20 CV of linear gradient with refolding buffer 1 (20 mM PBS, 500 mM NaCl, 1 mM dithiothreitol, 10 mM imidazole, pH 8.2), and another 10 CV of linear gradient with refolding buffer 2 (20 mM PBS, 500 mM NaCl, 10 mM imidazole, pH 8.2). The target protein was eluted with 10 CV of elution buffer (20 mM PBS, 500 mM NaCl, 350 mM imidazole, pH 7.8). All steps were run at a flow rate of 1 mL/min. Desalting of protein purified from IMAC was achieved using an Amicon Ultra–15 centrifugal filter unit (Millipore, MA) of 10 kDa NMWL. The retentate was diluted 1:2.5 with PBS buffer with glycerol 50% v/v.

The degree of purity of each MAb fragment was estimated from SDS-PAGE protein profiles using scanning densitometry analysis; Image J, an open source software, was used for image analysis.

### ELISA experiments

**Fig 3:**
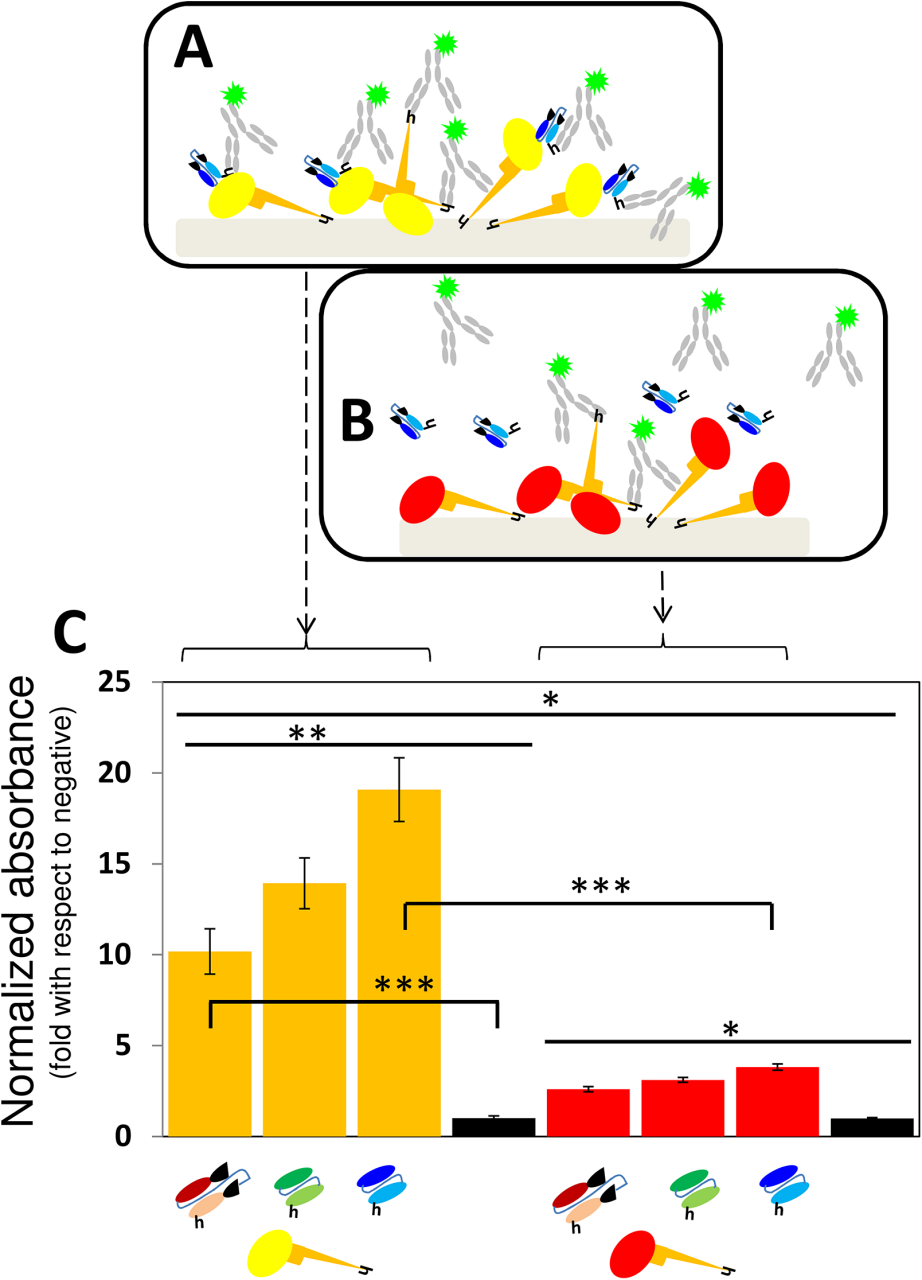
The binding of antibody fragments to GP: ELISA type I. (A) The specific binding of GP to Fab-KZ52, scFv-13C6, and scFv-13F6 was evaluated in ELISA experiments in which either rGP (yellow oval with orange stalk) or (B) HA-RBD (red oval with orange stalk) was attached to untreated assay surfaces. In these experiments, the binding between GP and the fragments was revealed using a solution of poly-clonal anti-histidine IgGs marked with horse radish peroxidase (gray Y). Negative controls consisted of surfaces with GP or HA-RBD not exposed to anti-GP fragments. (C) Plots of the absorbance signal, as measured in ELISA experiments, corresponding to assay wells where either GP (orange bars) or HA-RBD (red bars) was exposed to Fab-KZ52, (burgundy fragments), scFv-13F6 (green fragment), or scFv-13C6 (blue fragments). The absorbance signal has been normalized by the absorbance value of the negative controls (black bars). Symbols for mAb fragments are the same as those presented in Fig 1. Error bars indicate standard deviation from four repeats of independent ELISA experiments. Horizontal black lines indicate significant differences between groups (*p<0.05, **p<0.01; ***p<0.001).

In the first series of assays, a commercially available recombinant version of GP was attached to the surface of ELISA 96-well microplates and different full-length mAbs or antibody fragments were tested for specific binding (Fig 3A and 3B). For these experiments, 100 μL of a 0.5 μg/mL solution of His-tagged recombinant GP (rGP) expressed in insect cells (Integrated Biotherapeutics Bioservices; Gaithersburg, MD USA) were dispensed into each well, incubated for 12 h at 4°C, and washed twice with a 0.05% PBS-Tween solution. Afterwards, 200 μL of commercial blocking solution (Pierce; Rockford, IL USA) were dispensed per well, incubated at ambient temperature for 30 minutes, and then washed twice with PBS-Tween solution. The GP binding affinities of two different full-length monoclonal antibodies, namely mAb 13F6 (Integrated Biotherapeutics Bioservices; Gaithersburg, MD USA) and Infliximab (a commercial anti-rheumatoid arthritis therapeutic mAb from Janssen Biotech; Horsham, PA USA), and three antibody fragments (scFv-13C6, scFv-13F6, and Fab-KZ52) were then evaluated. For this purpose, 100 μL of a 1.0 μg/mL solution of each mAb or mAb fragment were dispensed into different wells, incubated for 1 h at room temperature, and washed three times with 0.05% PBS-Tween solution.

**Fig 4:**
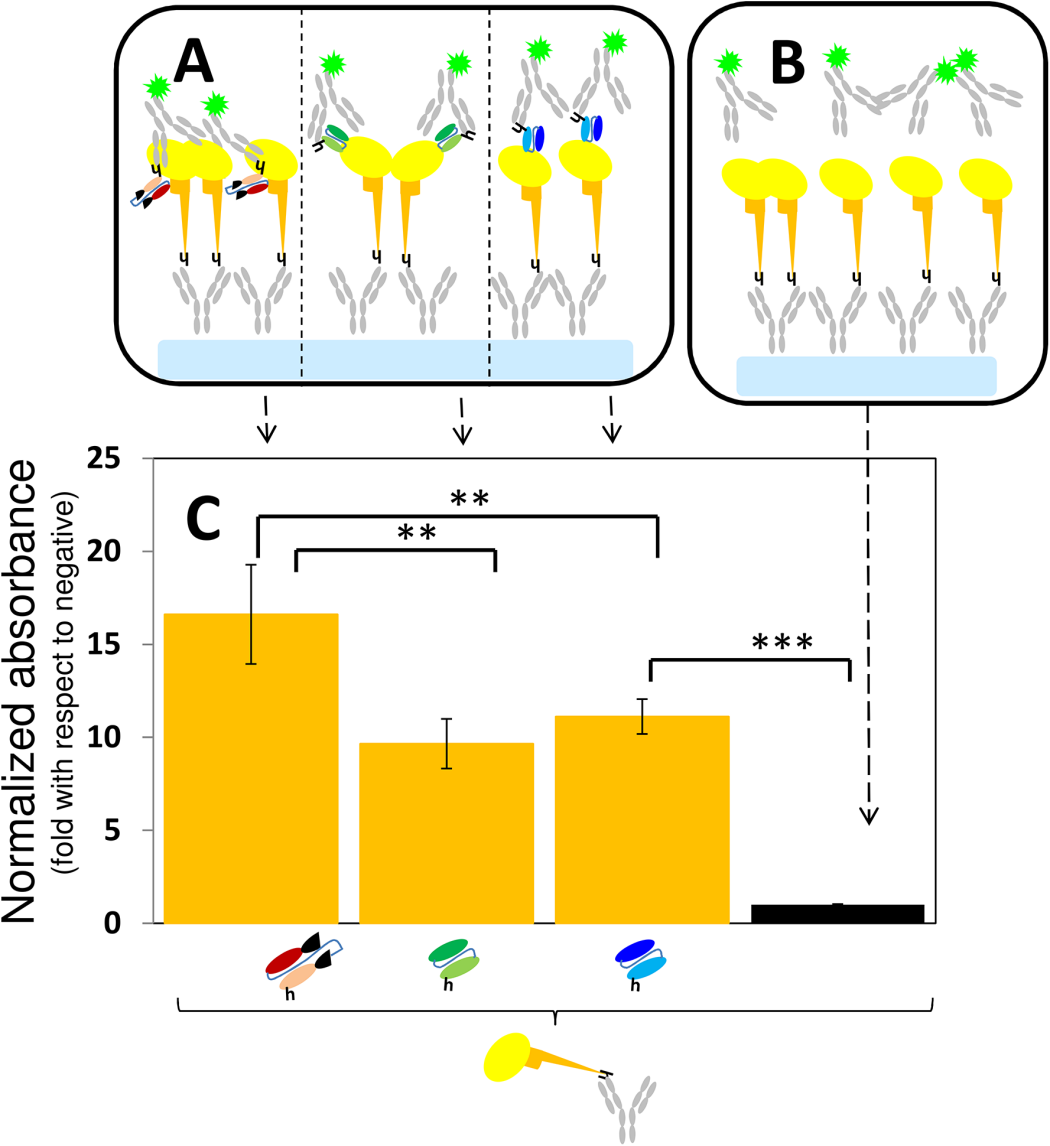
The binding of antibody fragments to GP: ELISA type II. (A) The specific binding of GP to Fab-KZ52, scFv-13C6, and scFv-13F6 was evaluated in ELISA experiments in which rGP (yellow oval with orange stalk) was attached to assay surfaces treated with anti-histidine IgGs (grey Ys). In these experiments, the binding between GP and the fragments was revealed using a solution of poly-clonal anti-histidine IgGs marked with horse radish peroxidase (grey Ys marked with a fluorescent star). (B) Negative controls consisted of surfaces with GP or HA-RGB not exposed to anti-GP fragments. (C) Plots of the absorbance signal, as measured in ELISA experiments, corresponding to assay wells where either GP (orange bars) was exposed to Fab-KZ52, (burgundy fragments), scFv-13F6 (green fragment) or scFv-13C6 (blue fragments). The absorbance signal has been normalized by the absorbance value of the negative controls (black bars). Symbols for mAb fragments are the same as those presented in Fig 1. Error bars indicate standard deviation from four repeats of independent ELISA experiments. Horizontal black lines indicate significant differences between groups (**p<0.01; ***p<0.001).

In a variation of this ELISA format (Fig 4A and 4B), 100 μL of either a 1 or 5 μg/mL solution of anti-Histidine Tag IgG (AbD serotec; Kidlington, UK) was dispensed into each well, incubated for 1 h at room temperature, and washed twice with a 0.05% PBS-Tween solution; 200 μL of commercial blocking solution were then dispensed per well, incubated at ambient temperature for 30 minutes, and then washed twice with 0.05% PBS-Tween solution. Afterwards, 100 μL of a 1 μg/mL solution of His-tagged rGP expressed in insect cells were dispensed into each well (or 100 μL of a 1 μg/mL solution of His-tagged HA-RBD expressed in-house) [38–40]; and incubated for 12 h at 4°C, and washed twice with a 0.05% PBS-Tween solution. The GP (or HA) binding affinities of two different full-length monoclonal antibodies (mAb 13F6) and antibody fragment Fab-KZ52 were then evaluated. Briefly, 100 μL of a 1.0 μg/mL solution of each mAb or mAb fragment were dispensed into different wells, incubated for 1 h at room temperature, and washed three times with 0.05% PBS-Tween solution.

In both ELISA formats, the GP-mAb complexes were revealed by adding 100 μL per well of a solution of an anti-human IgG-Fcγ marked with horseradish peroxidase (Pierce; Rockford, IL USA). The mAb fragments were revealed by adding a solution of anti-Polyhistidine IgG horseradish peroxidase conjugate (Sigma-Aldrich; St. Louis, MO USA). Revealing solutions were prepared in 0.05% PBS-Tween solution at dilution 1:120000 and 1:75000 respectively. Samples and revealing solutions were incubated for 1 h at room temperature, and then washed three times with PBS-Tween solution. Afterwards, 100 μL of TBM substrate (Pierce; Rockford, IL USA) were added per well and incubated for 15 minutes. Finally, 50 μL of a 1M H_2_SO_4_ solution was added to each well, and absorbance was measured at 450 nm in microplate reader (Biotek; Winooski, VT USA).

## Results and Discussion

### Expression of antibody fragments in bacterial cultures

We expressed a set of three anti-GP EBOV monoclonal antibody fragments in *E*. *coli* cultures. These fragments contained the variable regions (heavy and light) of three well-studied full-length mAbs referred in literature as 13C6, 13F6, and KZ52 (Fig 1A) [28]. Here, the corresponding mAb fragments are referred to as scFv-13C6, and scFv-13F6, and Fab-KZ52. The scFv-13C6 and scFv-13F6 proteins are single-chain variable fragments. The scFvs are the smallest stable antibody fragments capable of specifically binding an antigen. Generically, they are a recombinant polypeptide composed of an antibody variable light-chain amino acid sequence (VL) linked to a variable heavy-chain sequence (VH) by a designed peptide (of 10 to about 25 amino acids) that links the carboxyl terminus of the VL sequence to the amino terminus of the VH sequence. Fab-KZ52 was not strictly designed as an scFV, since it also contained portions of the constant heavy and constant light chains of full-length mAb KZ52 (see Fig 1B and 1C; Table 1).

Fig 2A shows the SDS-PAGE protein profiles of samples of bacterial suspensions of scFv-13C6, scFv-13F6, and Fab-KZ52 producer strains cultured in an instrumented 1.5 L bioreactor. We observed similar protein profiles and a similar degree of expression of the three fragments, as measured by SDS-PAGE gels and western blotting (Fig 2A and 2B). Cells were induced with IPTG at t = 8 h of culture. After induction, a band of protein of ≈30–50 KDa (30, 37, and 50 KDa for scFv-13C6, scFv-13F6, and Fab-KZ52 respectively) was observed, in agreement with the molecular weight of these fragments (Fig 2A and 2B). The additional bands in the Fab-KZ52 lane suggest the occurrence of a certain degree of fragmentation during culture.

Under the experimental conditions reported here, practically all the protein of interest was accumulated in inclusion bodies. The maximum biomass concentration in non-optimized batch cultures was 6–8 g/L and the product concentration after 18 h of culture was estimated at 20–80 mg/L.

After cultivation, each mAb fragment was recovered and purified using the protocols described in the Materials and Methods. An overall yield of ≈ 5–10 mg/L (mg of antibody fragment per L of culture media in the reactor) can be obtained by this non-optimized process with a final product purity higher than 98% (Fig 2C and 2D).

### ELISA experiments: rGP attached to the assay surface

**Fig 5:**
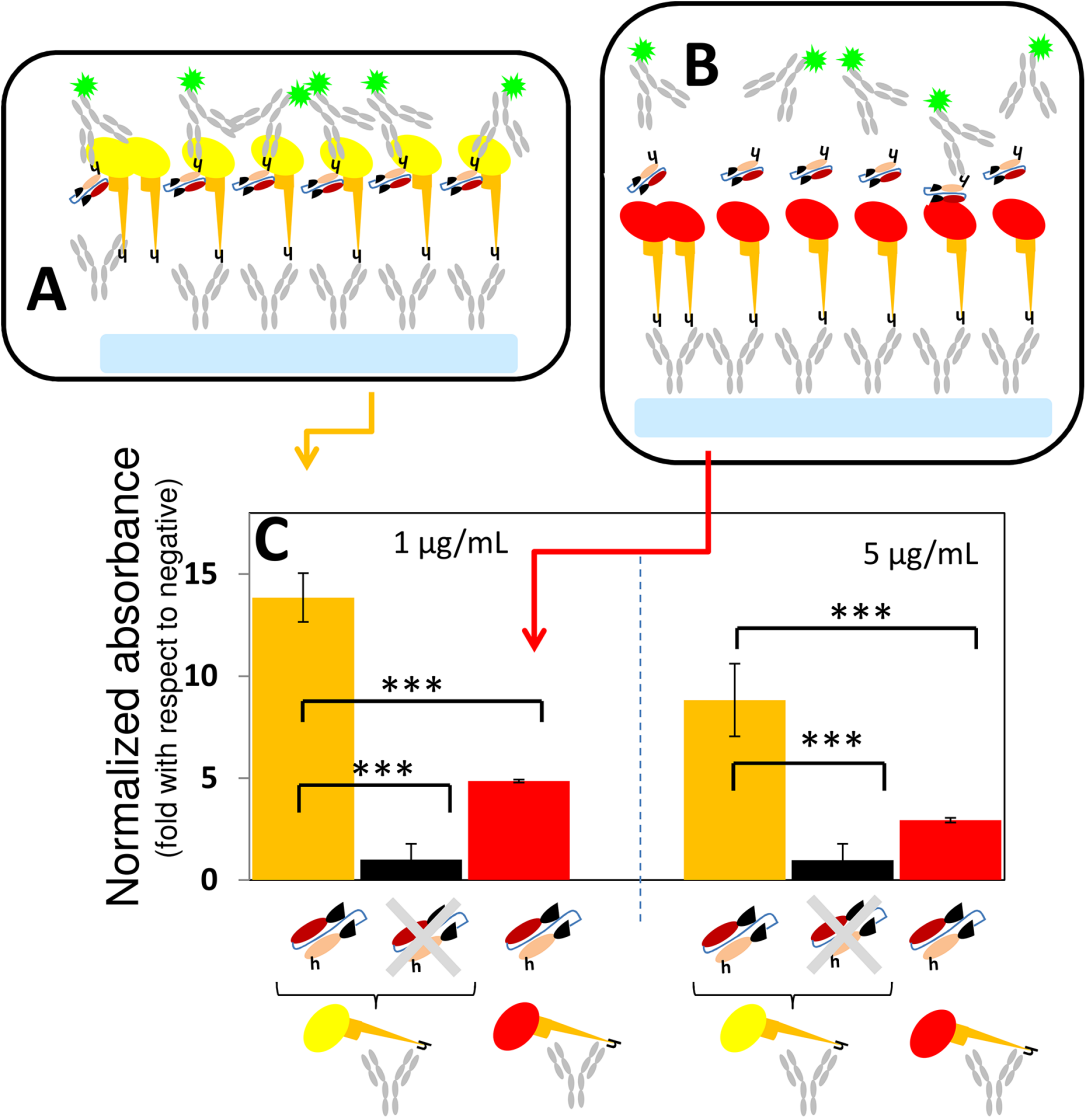
Binding activities of Fab-KZ52 to GP and HA-RBD in ELISA type II experiments. (A) The binding activity of Fab-KZ52 to GP was evaluated in ELISA experiments where a layer of anti-histidine IgGs (grey Ys) was first dispensed on the surface of assay wells for later attachment of a rGP layer (yellow ovals with orange stalk) (B) The non-specific binding of Fab-KZ52 to HA-RBD (red ovals with an orange stalk) was evaluated in a similar manner. (C) The binding activity in terms of absorbance units (normalized by the absorbance of the negative controls), as determined by ELISA experiments, of Fab-KZ52 to rGP (yellow bars), HA-RBD (red bars), and negative controls (black bars). Each panel shows results from experiments conducted at two different concentrations of the anti-histidine IgG solution (1 or 5 μg/mL) used to treat the assay surface. Symbols for mAb fragments are the same as those presented in Fig 1. Error bars indicate standard deviation from four repeats of independent ELISA experiments. Horizontal black lines indicate significant differences between groups (***p<0.001).

**Fig 6:**
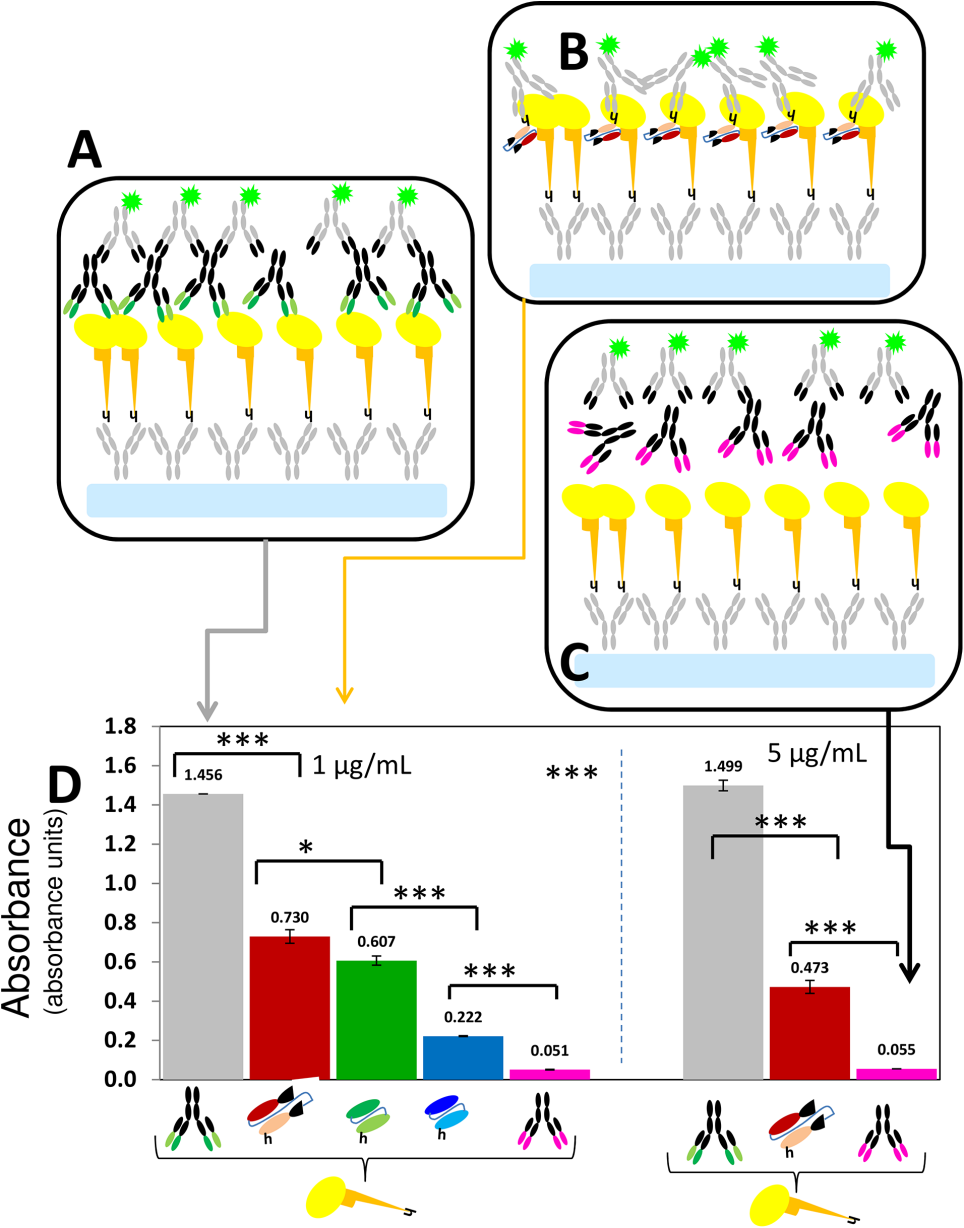
GP-binding activities of mAb-13F6, Fab-KZ52, scFv-13F6, scFv-13C6 in ELISA type II experiments. (A) The binding activity of full-length mAb-3F6 to GP was evaluated in ELISA experiments, where a layer of anti-histidine IgGs (grey Ys) was first dispensed on the surface of assay wells for later attachment of a rGP layer (yellow ovals with orange stalks). The presence of the mAb+GP complex was revealed by the addition of a solution of anti-IgG polyclonal-IgGs (grey Ys with black variable regions and green fluorescent stars). (B) Analogously, the binding activity of Fab-KZ52, scFv-13F6, and scFv-13C6 to GP was evaluated in ELISA experiments where a layer of anti-histidine IgGs was first dispensed on the surface of assay wells (grey Ys) for later attachment of an rGP layer (yellow ovals with orange stalks). The presence of the mAb fragment+GP complexes was revealed by the addition of a solution of anti-histidine polyclonal-IgGs (grey Ys with green fluorescent stars). (C) Negative control experiments consisted of the use of Infliximab (anti-rheumatoid arthritis therapeutic mAb; black mAb with pink variable regions) instead of an anti-GP mAb or fragment. (D) Binding activity (in terms of arbitrary absorbance units), as determined by ELISA experiments of the full-length mAbs-13F6 (black mAb with green variable regions), Fab-KZ52 (burgundy fragment), scFv-13F6 (green fragment), and scFv-13C6 (blue fragment) to GP (yellow ovals with orange stalks). The absorbance reading of negative controls are also shown (pink bars). Each panel shows results from experiments conducted at two different concentrations of the anti-histidine IgG solution (1 or 5 μg/mL) used to treat the assay surface. Error bars indicate standard deviation from at least two repeats of independent ELISA experiments. No normalization has been performed. Horizontal black lines indicate significant differences between groups (*p<0.05, **p<0.01; ***p<0.001).

The binding capacity of each antibody fragment (Fab-KZ52, scFv-13C6, and scFv-13F6) to a commercial recombinant EBOV GP (rGP) expressed in insect cells was determined in ELISA experiments (Figs 3–6). In a first round of experiments, rGP (commercial) was attached to assay well plate surfaces and either full length mAb 13F6 (positive control), scFv-13F6, scFv-13C6, or Fab-KZ52 was dispensed at a concentration of 0.5 μg/mL. Assays with the HA-RBD from Influenza A/H1N1/2009 attached to well surfaces were used as negative controls. In these experiments, mAb 13F6, Fab-KZ52, scFv-13F6, and scFv-13C6 were confirmed to have attached to the surface functionalized with rGP as revealed by the addition and binding of commercial rabbit polyclonal anti-IgGs (in the case of experiments using fragments) and poly-clonal anti-constant region IgGs (in the case of experiments using mAb 13F6).

All three antibody fragments exhibited selective GP binding activity in ELISA experiments, but significant differences in binding activity and selectivity were found among them. Fig 3C shows a comparison of the binding activity against GP (orange columns) as determined in ELISA assays, where GP is attached to the assay surface. The scFv-13C6 mAb exhibited the highest GP-binding activity, followed by scFv-13F6 and Fab-KZ52. The absorbance signal ratio between positive binding samples and blanks (no fragment added) was 10.18(±1.246), 13.93(±1.397), and 19.08(±1.750) for Fab-KZ52, scFv13F6, and scFv-13C6, respectively. We run additional negative controls, using human serum—from Mexican patients exposed to Influenza A/H1N1/2009 and presumably negative for Ebola—instead of PBS. These negative controls exhibit a similar absolute absorbance signal to that of a PBS sample.

Red columns indicate the absorbance signal relative to the blank (experiments where no antibody-fragment added) when another virus capsid protein, HA-RBD from Influenza A/H1N1/2009, was attached to the assay surface instead of GP. The ratio of GP-binding affinity to HA-RBD affinity (GP/HA), an indicator of selectivity, was 3.91(±0.468), 4.48(±0.439), and 5.00(±0.455) for Fab-KZ52, scFV-13F6, and scFv-13C6, respectively.

Although fragment Fab-KZ52 did bind to GP, its affinity and selectivity were the lowest among the three fragments tested. A probable explanation for this could be the nature of the KZ52 epitope. While the conformational epitopes for 13C6 and 13F6 only include GP1 domain residues [33], KZ52 (and its analogous fragment Fab-KZ52) should interact with residues at GP1 and GP2 for proper binding. Any misalignment of the variable regions in the fragment could conceivably cause a decrease in binding efficacy [41]. In addition, the Fab-KZ52 epitope is less exposed than the scFv-13C6 or scFv-13F6. Since the access to the KZ52 epitope is sterically more impaired (not shown), the proper alignment of GP molecules could favor the access of antibody fragments to it. We tested this hypothesis by conducting binding experiments using a slightly modified ELISA format in which a layer of anti-histidine IgGs was dispensed at the surface of the assay wells for later addition of his-tagged rGP. While the direct attachment of rGP to the assay surface does not favor any particular alignment of the molecule, the layer of anti-histidine antibodies induces a preferred orientation for the His-tagged GP (Fig 4A) and HA-RBD molecules. Indeed, further improvement in the performance of ELISA Fab-KZ52 experiments was achieved by using an anti-histidine IgG layer (Fig 4C). The average Fab-KZ52 GP binding affinity (normalized by the negative control) increased 65.00%(±29.94%), from 10.18(±1.246) (Fig 3C) to 16.61(±2.674) (Fig 4C). Interestingly, the average GP-binding affinity of the other two fragments (scFv-13C6 and scFv-13F6) decreased. On average, the apparent scFv-13F6 GP binding affinity decreased 30.20%(±10.69%) (from 13.93(±1.397) to 9.65(±1.336) normalized units) and the apparent scFv-13C6 GP binding affinity decreased by 41.34%(±6.54)% (from 19.08(±1.750) to 11.12(±0.938) normalized units) (see Figs 3C and 4C). Taken together, these results suggest that the effects of molecular orientation and alignment are relevant in the design of strategies for the diagnosis, capture, or blockage of EBOV. They also suggest that it may be useful to include more than one antibody fragment (actually a cocktail of them) to maximize binding.

In this system, steric effects appear to be important at the molecular level. This has implications for the design of protocols to treat/sensitize surfaces in ELISA methods. Fig 5A–5C shows results from Fab-KZ52 + GP ELISA experiments that evaluated two different anti-histidine IgG surface densities associated with the use of two different anti-histidine IgG coating solution concentrations (1 or 5 μg/mL). The non-specific binding of Fab-KZ52 to HA-RBD was also measured (Fig 5B). The sensitivity of the assay was higher for the surface with lower anti-histidine IgG density. On average, the ratio of absorbance signals between positive samples (Fab-KZ52 + GP) and negative controls was 13.86(±1.053) and 9.03(±0.337) units, respectively, in assays with surfaces treated with 1 and 5 μg/mL of anti-histidine IgGs (Fig 5C). This ratio increased to 16.61(±2.674) units in assays conducted on surfaces treated with 0.5 μg/mL of anti-histidine IgGs (Fig 4C). The Fab-KZ52 selectivity, measured as the ratio of the absorbance signals between GP and HA-RBD binding assays, did not increase significantly in the experiments using assay surfaces treated with 1 and 5 μg/mL of anti-histidine IgGs (GP/HA = 3.32(±0.121) and 4.03(±0.153), respectively) when compared with the experiments conducted with GP directly attached to the assay surface (GP/HA = 3.91(±0.468)).

In our experiments, we also included full-length mAb 13F6 and Infliximab (a commercial anti-rheumatoid arthritis therapeutic mAb) as a positive and negative control, respectively (Fig 6A–6C). On average, the ratio of absorbance signals due to GP&mAb-13F6 and GP&Fab-KZ52 binding was 2.00(±0.161) and 3.187(±0.269) in experiments using 1 and 5 μg/mL of anti-histidine IgG coating solution, respectively (Fig 6D). These results are provided only as a reference to indicate the level of sensitivity expected when using a an anti-GP(EBOV) fragment instead of a commercially available full-length mAb. They should not be considered useful for a direct comparison of the binding affinities of full-length mAbs and antibody fragments. Note that MAb 13F6 and Fab-KZ52 target different epitopes at GP. We also revealed the GP&mAb-13F6 complex by using a marked poly-clonal IgG cocktail instead of anti-histidine IgGs (data not shown). We also present ELISA results derived from the use of scFv-13F6, and scFv-13C6 (Fig 6D). The three fragments produced and tested exhibited significant differences in binding affinity to GP, but all of them in the same order of magnitude.

EBOV GP binding does not absolutely guarantee that these three mAb fragments will be effective in detecting EBOV particles in patient samples. Research using samples containing actual EBOV particles and samples from Ebola disease patients must be conducted to fully validate the diagnostic usefulness of these peptides.

## Conclusions

We show that three antibody fragments (designated Fab-KZ52, scFv-13C6, and scFv-13F6, to correspond to the full-length mAbs from which their variable regions were taken) are able to bind a commercially available GP with comparable affinity (lower but in the same order of magnitude) to that seen with the previously studied and now commercially available full length anti-GP mAbs. This finding is of substantial relevance to the design of GP detection methods (and even therapeutic strategies). The use of antibody fragments rather than full length antibodies will significantly reduce the cost of production of Ebola diagnostic devices. The production of mAb fragments in bacterial cultures is a more cost-effective proposition than the production of full-length mAbs in insects or mammalian cell cultures.

We evaluated the binding affinity of GP to each one of these three fragments in ELISA experiments in which a commercial his-tagged rGP was either directly attached to the assay surface or attached via a layer of anti-histidine IgGs. All three fragments bound GP. Our results suggest that the apparent sensitivity of the assay depends on the orientation/alignment of rGP molecules at the assay surface. We also found that the concentration of the anti-histidine solution used to prepare the surface for rGP binding is relevant for the sensibility and selectivity of the assay.

Antibody fragments can be a useful addition to the toolbox for diagnosis of the presence of GP in biological samples. They may allow the design of less expensive and high-load/high resolution EBOV detection systems. For instance, nanoparticles highly loaded with antibody fragments could represent a cost-effective alternative for the detection and capture/concentration of GP and EBOV particles.

In a more general sense, deriving functional mAb fragments from well-studied full length mAbs, and producing these fragments in bacterial culture, might be a cost-effective (and expeditious) strategy for reacting to diagnostic needs in epidemic (or even pandemic) events. We are offering a first proof-of-principle for the particular case of Ebola.
